# Role of Tyr-39 for the Structural Features of α-Synuclein and for the Interaction with a Strong Modulator of Its Amyloid Assembly

**DOI:** 10.3390/ijms21145061

**Published:** 2020-07-17

**Authors:** Oscar Palomino-Hernandez, Fiamma A. Buratti, Pamela S. Sacco, Giulia Rossetti, Paolo Carloni, Claudio O. Fernandez

**Affiliations:** 1 Computational Biomedicine, Institute for Neuroscience and Medicine (INM-9) and Institute for Advanced Simulations (IAS-5), Forschungszentrum Jülich, 52425 Jülich, Germany; o.palomino@fz-juelich.de (O.P.-H.); g.rossetti@fz-juelich.de (G.R.); 2 Faculty of Mathematics, Computer Science and Natural Sciences, RWTH Aachen, 52425 Aachen, Germany; 3 Computation-Based Science and Technology Research Center, The Cyprus Institute, 2121 Nicosia, Cyprus; 4 Institute of Life Science, The Hebrew University of Jerusalem, 91904 Jerusalem, Israel; 5 Max Planck Laboratory for Structural Biology, Chemistry and Molecular Biophysics of Rosario (MPLbioR, UNR-MPIbpC) and Instituto de Investigaciones para el Descubrimiento de Fármacos de Rosario (IIDEFAR, UNR-CONICET), Universidad Nacional de Rosario, S2002LRK Rosario, Argentina; buratti@iidefar-conicet.gob.ar (F.A.B.); sacco@iidefar-conicet.gob.ar (P.S.S.); 6 Department of Oncology, Hematology, Oncology, Hemostaseology, and Stem Cell Transplantation University Hospital Aachen, RWTH Aachen University, Pauwelsstraße 30, 52074 Aachen, Germany; 7 Jülich Supercomputing Center (JSC), Forschungszentrum Jülich, 52425 Jülich, Germany; 8 Institute for Neuroscience and Medicine (INM-11) Forschungszentrum Jülich, 52425 Jülich, Germany; 9 Department of NMR-Based Structural Biology, Max Planck Institute for Biophysical Chemistry, Am Fassberg 11, D-37077 Göttingen, Germany

**Keywords:** alpha synuclein, mutagenesis, aromaticity, aggregation, molecular simulation

## Abstract

Recent studies suggest that Tyr-39 might play a critical role for both the normal function and the pathological dysfunction of α-synuclein (αS), an intrinsically disordered protein involved in Parkinson’s disease. We perform here a comparative analysis between the structural features of human αS and its Y39A, Y39F, and Y39L variants. By the combined application of site-directed mutagenesis, biophysical techniques, and enhanced sampling molecular simulations, we show that removing aromatic functionality at position 39 of monomeric αS leads to protein variants populating more compact conformations, conserving its disordered nature and secondary structure propensities. Contrasting with the subtle changes induced by mutations on the protein structure, removing aromaticity at position 39 impacts strongly on the interaction of αS with the potent amyloid inhibitor phthalocyanine tetrasulfonate (PcTS). Our findings further support the role of Tyr-39 in forming essential inter and intramolecular contacts that might have important repercussions for the function and the dysfunction of αS.

## 1. Introduction

Neurodegenerative disorders such as Parkinson’s and Alzheimer’s are among the human diseases associated with the self-assembly of polypeptides into amyloid structures [1,2]. Toward the design of effective therapeutics to combat these diseases, one of the major unanswered questions of protein aggregation is the propensity of particular primary sequences to aggregate [1,2]. These relevant regions are usually known as aggregation “hot spots”, and their study becomes critical to identify determinants governing protein aggregation and to reveal specific interactions that must be disrupted to prevent amyloid assembly. This knowledge can in turn be used for therapeutic intervention to target the aggregation pathway of these proteins and its associated toxicity [3].

The π-stacking of aromatic residues has been suggested to serve as structural and functional elements for the progression of the self-assembly process [4]. Furthermore, the role of aromatic–aromatic interactions in aggregation was also suggested by an examination of compounds that inhibit fibril formation [5]. Aromatic groups are a frequent feature in these inhibitors [6,7,8,9,10,11,12,13,14,15,16,17,18,19,20,21,22,23,24]; hence, the ability of these molecules to impair amyloid formation might be mediated by π−π interactions with aromatic residues in specific regions of the protein sequence. 

In that context, the implication of Tyr residues on the aggregation of the amyloid protein alpha synuclein (αS), a pathological factor in Parkinson’s disease, has been explored by mutagenesis-based approaches [25,26,27,28,29]. In its monomeric, intrinsically disordered state, αS adopts an ensemble of conformations with no rigid secondary structure. However, long-range interactions have been shown to stabilize an aggregation-autoinhibited conformation. The protein contains three Tyr residues in the C-terminal region (Tyr-125, Tyr-133, Tyr-136) and one in the N-terminus (Tyr-39). Cysteine substitution in the latter but not in Tyr-125, Tyr-133, and Tyr-136 positions preferentially enhanced dimer and oligomer formation under oxidative conditions, suggesting a dimerization pathway mediated by this region [25]. On the other hand, substitution of Tyr-39 and Tyr-133 by Ala resulted in substantial inhibition of fibrillation, suggesting that Tyr-39 and the C-terminal Tyr residues might form an aromatic cluster that stabilizes the native state of αS [26]. More recently, the structural elucidation of the inhibitory interaction between αS and phthalocyanine tetrasulfonate (PcTS), a widely used anti-amyloid compound, provided the basis for the rational design of αS variants, indicating that the aromatic side chain of the Tyr residue at position 39 might have a critical role in the fibrillation pathway of αS [5,6]. Phthalocyanines such as PcTS are small molecules inhibiting the formation of fibrillar aggregates for targets such as αS, tau, and prion protein. The aromatic character of these molecules was proposed as a key feature to enable inhibitory interactions with these proteins [5].

Substituting Tyr by Ala [6,26] or Cys [25] not only replaces an aromatic side chain with an aliphatic one, but it also affects considerably the hydrophobicity, the size, and the β-sheet propensity of the side chain. To uncover the role of aromaticity at position 39 for aggregation, here we replaced Tyr-39 with the following residues: (i) Phe, which keeps aromaticity and removes a hydrophilic group such as OH; (ii) Leu, which is similar to Phe in size and hydrophobicity but it is not aromatic; (iii) Ala, which is hydrophobic as Phe and Leu but it is smaller in size. By using a combined approach based on experimental biophysics and replica exchange with solute tempering (REST2) enhanced sampling molecular simulations [30], we analyzed the impact of these mutations on monomeric αS’ structural properties and on the interactions of the protein with PcTS. The structural changes induced by the mutations turned out to be only local, with the monomeric state of αS remaining disordered upon replacement of Tyr-39 by Phe, Leu and Ala. Moreover, the four αS species showed no propensities for specific elements of secondary structure, adopting in all cases typical random-coil conformations. Interestingly, our simulations suggest that the conformations populated by the Y39A and the Y39L variants were more compact than Y39F and wild-type (*wt*) species. Aromaticity at position 39 resulted in a critical structural determinant for PcTS binding to the N-terminus of αS, an event that mediates the inhibition of this molecule on αS fibril assembly. Overall, these new findings give further support to a role for Tyr-39 in essential inter and intramolecular contacts that might have important repercussions for the function and the dysfunction of αS.

## 2. Results

### 2.1. Monomeric αS Remains Disordered after Y39 Mutation

**Figure 1:**
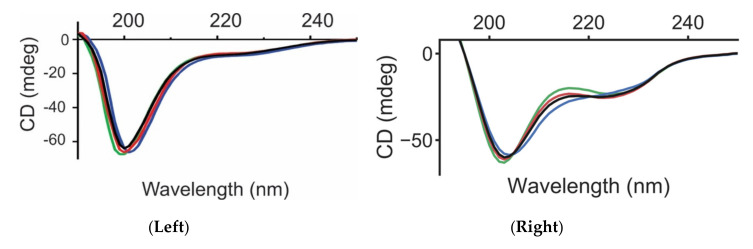
The position-39 αS variants are largely disordered in the monomeric state. The far-UV circular dichroism (CD) spectra of *wt* (black), Y39F (blue), Y39A (red), and Y39L (green) αS monomers show spectral features that are characteristic of random coil conformations (**Left**). In comparison, the predicted CD from the molecular ensemble for the four variants is also shown (**Right**).

The structural features of the monomeric wild-type (*wt*), Y39F, Y39L, and Y39A αS species were investigated by circular dichroism (CD), NMR spectroscopy, along with REST2 simulations [30]. The latter allows one to enhance the sampling of the conformational space of the protein relative to plain molecular dynamics (MD) [30]. As determined for the wild-type protein, the far-UV CD spectra of all single mutants at position 39 were indicative of largely disordered conformations [6], as manifested in prominent negative minima at ~199 nm (Figure 1 Left). The predicted CD spectra of the four simulated variants were consistent with the experimental ones (Figure 1 Right). In particular, it suggests that random coil is the most predominant motif on the *wt* and the three variants.

**Figure 2:**
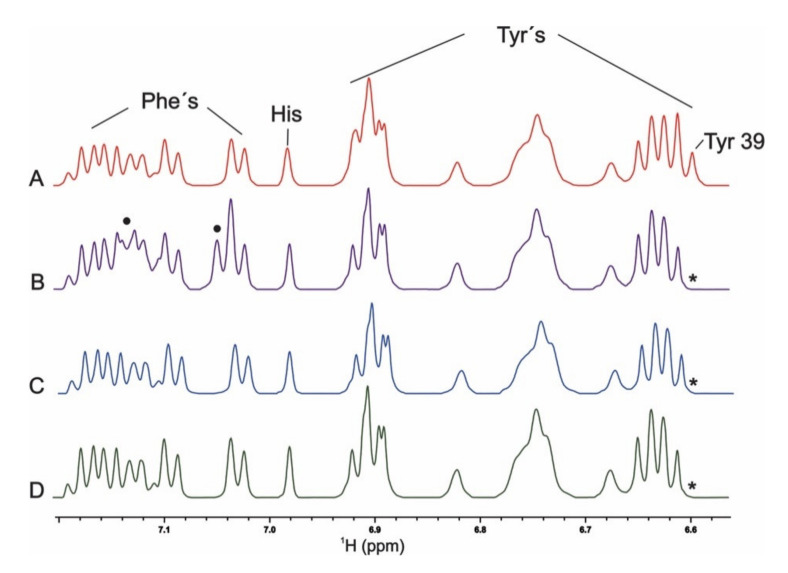
^1^H NMR of aromatic side chains of the position-39 αS proteins. Spectra were registered at 15 °C in Buffer A of samples containing 100 µM *wt* (**A**), Y39F (**B**), Y39A (**C**), and Y39L αS (**D**). Asterisks indicate the lack of peaks corresponding to the Tyr-39 residue. Circles indicate the detection of new signals assigned to the Phe residue in position 39.

The NMR spectra of non-labeled *wt* αS in Buffer A showed well-resolved clusters of resonances in the 6.0–8.0 ppm (^1^H) range, comprising the side chains of different aromatic residues: His (aa50), Phe (aa4, aa94), and Tyr (aa39, aa125, aa133, aa136) (Figure 2) [6,31]. The replacement of tyrosine at position 39 by phenylalanine, leucine, and alanine residues in the 1D ^1^H NMR spectra of the mutant species was confirmed by the lack of signals corresponding to the Tyr-39 residue (Figure 2B–D) and the detection of new signals corresponding to the presence of an additional phenylalanine residue in the Y39F variant (Figure 2B). 

**Figure 3:**
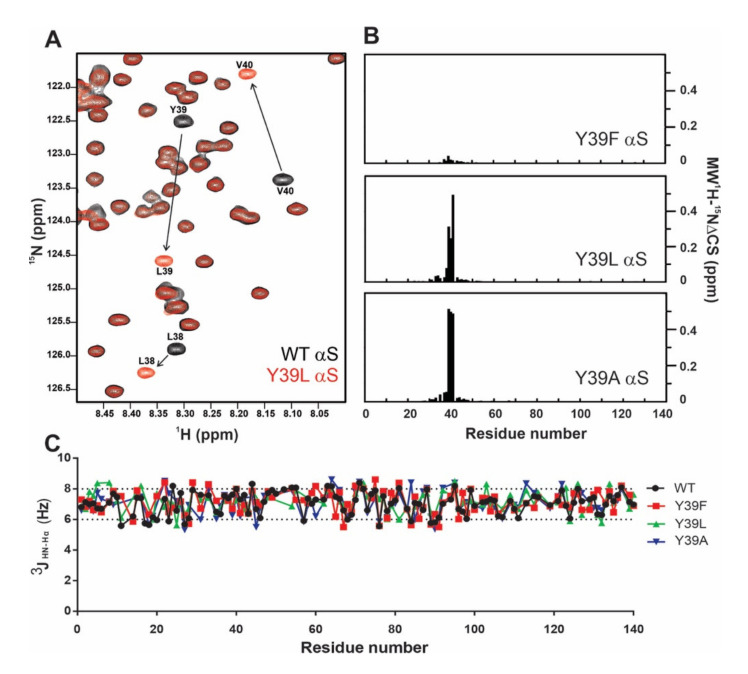
Changes induced by the mutations are only local and restricted to the immediate vicinity of the position-39 residue. (**A**) Overlaid contour plots of ^1^H-^15^N heteronuclear single quantum correlation (HSQC) spectra of 50 µM *wt* (black) and Y39L αS (red). Most affected residues located in the vicinity of the mutated site are labeled. (**B**) Differences in the mean weighted chemical shifts (MW ^1^H-^15^N ΔCS) displacements between *wt* and Y39F, Y39A, and Y39L αS mutants. (**C**) Panel shows the ^3^J_HN-Hα_ profiles measured for the *wt* and Y39F, Y39A, and Y39L αS proteins. In all cases, experiments were recorded at 15 °C using ^15^N isotopically enriched protein samples (50 µM) dissolved in Buffer A.

The overlaid ^1^H-^15^N heteronuclear single quantum correlation (HSQC) spectra of a 50 µM sample of uniformly ^15^N-labeled *wt* αS and its Y39L variant recorded in Buffer A at 15 °C is shown in Figure 3A. The resonances were well resolved and sharp, with a poor dispersion of chemical shifts, reflecting the disordered nature of the backbone (Figure S1). As shown in Figure 3B, changes induced by the mutations were only local and were restricted to the immediate vicinity of the Tyr residue. The absence of noticeable changes in the structural properties indicated that the monomeric state of the protein variants remained disordered upon replacement of Tyr-39 by Phe, Leu, and Ala.

### 2.2. Structural Propensities of the Y39 αS Variants

We also evaluated the different protein variants in terms of their conformational properties. ^3^J_HN-Hα_ couplings are reliable quantitative reporters of the time-averaged distribution of the backbone torsion angles φ and are frequently used to probe the propensity of intrinsically disordered proteins (IDP) to sample different regions of conformational space [32]. Therefore, we measured residue-specific ^3^J_HN-Hα_ couplings in all of these proteins. As shown in Figure 3C, the values measured for the *wt* and Y39F, Y39L, and Y39A αS proteins were typical of random-coil conformations and essentially indistinguishable among the mutant species studied. 

The determination of the hydrodynamic properties of a macromolecule was extensively applied in the study of conformational changes accompanying processes such as molecular association and folding [33,34]. We then employed pulse field gradient-NMR (PFG-NMR) to measure the hydrodynamic properties of *wt* αS and its mutant variants at position 39. The values of the hydrodynamic radius (R_H_) determined for the protein in its native state (R_H_ = 31.0 ± 0.4 Å) were consistent with previous determinations [31,35], whereas no changes were detected in the Y39F variant (R_H_ = 30.9 ± 0.4 Å). Compared with the parameters measured for the Y39A (R_H_ = 28.0 ± 0.4 Å) and the Y39L (R_H_ = 28.4 ± 0.4 Å) mutants, our results suggest that the latter mutations might affect the size of native αS or cause some collapse to a more compact species. The small percentage deviation across the R_H_ values of the four αS species allowed us to detect differences in the compactness of the protein. This was shown by the following facts: (i) the measured R_H_ values of αS in its native state (31.9 Å) and in the presence of 8 M urea (35.0 Å) reflected the decreased persistence of the residual long-range interactions in αS, as demonstrated conclusively by paramagnetic relaxation enhancement measurements [36]. Thus, the change of ~10% (3.1 Å) in R_H_ was significant and consistent with the transition of αS from its native state towards an ensemble of more extended, unfolded conformations. (ii) The measured R_H_ value of αS in dilute solution (26.6 ± 0.5 Å) changed to 22.5 ± 0.6 Å in 1 M glucose. The decrease of ~15% (4 Å) was consistent with the fact that the protein adopts more compact states under crowding conditions [37,38].

In addition to the previous results, we expressed the compactness of the protein in terms of “compaction factors”, which relate the measured R_H_ with empirical estimations of minimum and maximum values expected for the length of the polypeptide chain for each conformational ensemble of a protein [36,39,40]. The values ranged from 1.0 for a natively folded protein to 0.0 for a pure random coil. The calculated compaction factors turned out to be 0.30 for *wt* and for Y39F αS, consistently with previously published results [36]. The compaction factors were instead 0.50 both for Y39L αS and Y39A αS. Such an increase of ~65% in compaction factor values was significantly indicative of a higher degree of compaction for Y39A and Y39L relative to *wt* αS. We concluded that the Y39A and the Y39L protein variants adopted an ensemble of more compact conformations in solution than the Y39F and the *wt* proteins.

**Figure 4:**
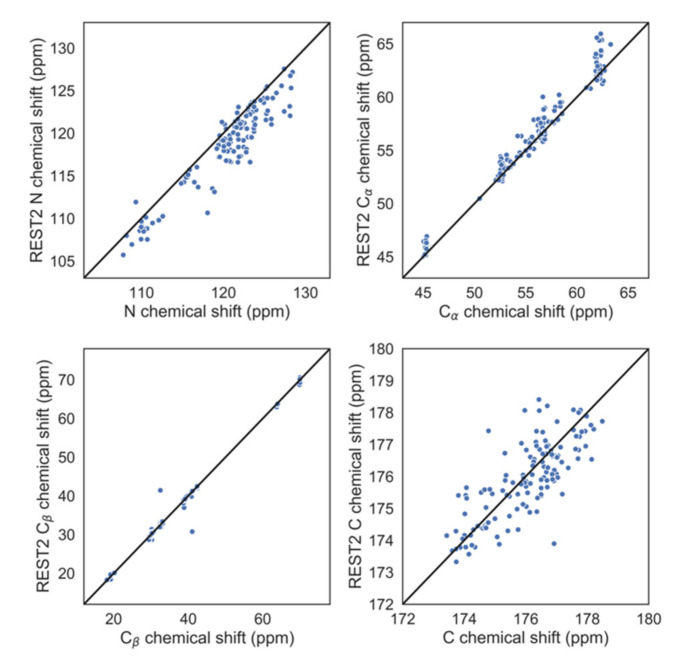
Predicted versus experimental chemical shifts of *wt* αS. Scatterplots showing the comparison between experimental chemical shifts of C, C, Cβ, and N atoms and those calculated from our replica exchange with solute tempering (REST2) simulations using the SHIFTX2 code (see Methods for details).

The REST2 simulations were validated through comparison with experimental data. The predicted chemical shifts N, Cα, and Cβ atoms of *wt* correlated well with the corresponding experimental values, while the C atoms had more outliers, making the correlation only fair (Figure 4). The *wt* protein exhibited partial (10%) α-helical propensities within its first ten residues (Figure S2), consistently with the experiment [41]. The REST2 simulations suggested that the four αS species shared similar levels of secondary structure. Indeed, the content of secondary structure did not change significantly on passing from the *wt* to the three mutants investigated here (Table 1). However, the latter exhibited a different local organization of the secondary structure’s motifs (Figure S2). These results are in agreement with the above reported ^3^J_HN-Hα_ profiles, indicating that changes induced by the mutations were only local and were restricted to the immediate vicinity of the position-39 residue.

**Figure 5:**
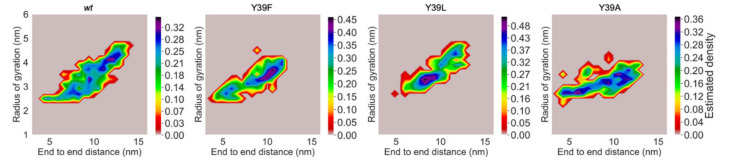
Conformational preferences observed for *wt* αS and its Y39A, Y39F, and Y39L mutants. Density maps of the radius of gyration (RG) and end-to-end (EE) distance reaction coordinates for the simulated structural ensembles.

The compactness of the proteins was further investigated through a projection of the radius of gyration (RG) and the end-to-end distance (EE) as reaction coordinates, as performed for other IDP analyses [42]. Figure 5 shows that *wt* and Y39F shared a similar compactness profile. They populated mostly regions of the conformational space with relatively large RG and EE, therefore having preference for more expanded structures. In contrast, Y39L and Y39A mostly populated regions with progressively lower values of RG and EE (additional information on the minima is reported in Table S1). At the qualitative level, this suggested the following trend in compactness: Y39A > Y39L > Y39F ~ *wt*, which is consistent with the experimental results (vide supra). 

We next analyzed the proteins intramolecular hydrogen bonds (HB) and hydrophobic contacts across the simulations of the four species. 

**Figure 6:**
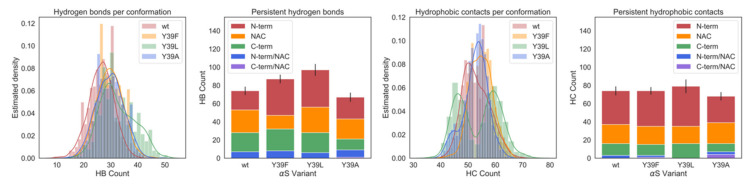
Hydrogen bonds (HB) and hydrophobic contacts (HC) of the four species investigated here. First panel from the left: distribution of HB contacts, with average values of 26.5, 29.6, 32.4, and 29.6 for *wt*, Y39F, Y39L, and Y39A αS, respectively. Second panel: HB contacts in each protein region. An HB contact was assumed to be present if persistent for 8% of the simulation or longer. Third panel: Distribution of HC contacts, with average values at 53.0, 54.8, 53.8, and 52.7 for *wt*, Y39F, Y39L, and Y39A αS, respectively. Fourth panel: HC contacts in each protein region. The HC contacts were assumed to be present if persistent for 15% of the simulation or longer. In the second and the fourth panels, error bars are reported. They were computed from the standard deviations of the distributions in the first and the third panels, respectively.

Overall, Y39L and Y39F αS exhibited more HB contacts than the *wt*, with significant changes in the non-amyloid-β component (NAC) region (Figure 6 and Table S2). Instead, the number of salt-bridges is similar across the four proteins (data not shown). As far as the position 39 was concerned, we found that Y39 side chain in *wt* was mostly solvent exposed, with an average of ~1.7 HBs with the solvent during the simulation. It formed intramolecular HB contacts (with K21/G25/T44 backbone units) for only 2% of the simulated time. The backbone unit of the residue across the four species was also mostly solvated. It formed HB contacts with the backbone units of neighboring residues for only less than 10% of the simulated time. We concluded that the content of intramolecular HB contacts was rather small, albeit it varied across the four species (Figure 6).

The overall number of hydrophobic contacts increased on passing from *wt* to Y39F and Y39L αS, while it decreased on passing from *wt* to Y39A αS (Figure 6 and Table S3). However, while the first three species formed only intra-region contacts, Y39A αS also displayed inter-region contacts. The residue in position 39 formed significant interactions with its neighboring residues; it interacted with (i) L38 for almost the entire simulation time across the four species; (ii) V40 for ~50 to ~70% of simulated time on passing from *wt*/Y39A to Y39F and Y39L αS; (iii) both G47 and V48 for ~55% of the simulated time in Y39A αS, ~70% in Y39L αS/*wt*; ~90% in Y39F αS.

We concluded that the intramolecular contacts of residue in position 39 across the four species involved the N-term residues, and they were basically hydrophobic in nature. Indeed, as pointed out above, the HB contacts of the residue in position 39 are formed mostly with the solvent across the four species. These contacts may have played a role for the observed increase of compactness on passing from *wt* and Y39F αS to the other two mutants (Figure 5). On one hand, Y39L αS showed a higher number of hydrophobic contacts than Y39F αS and *wt* (Figure 6). This might have contributed to the formation of a more compact structure. On the other hand, as already mentioned above, Y39A αS was the only species out of the four investigated here forming significant inter-region residue hydrophobic contacts. The latter may have been important for increasing the compactness of the protein. The difference between Y39L and Y39A variants may have been caused, at least in part, by the different conformational properties of the two residues [43].

### 2.3. Aromaticity at Position 39 and PcTS Binding

**Figure 7:**
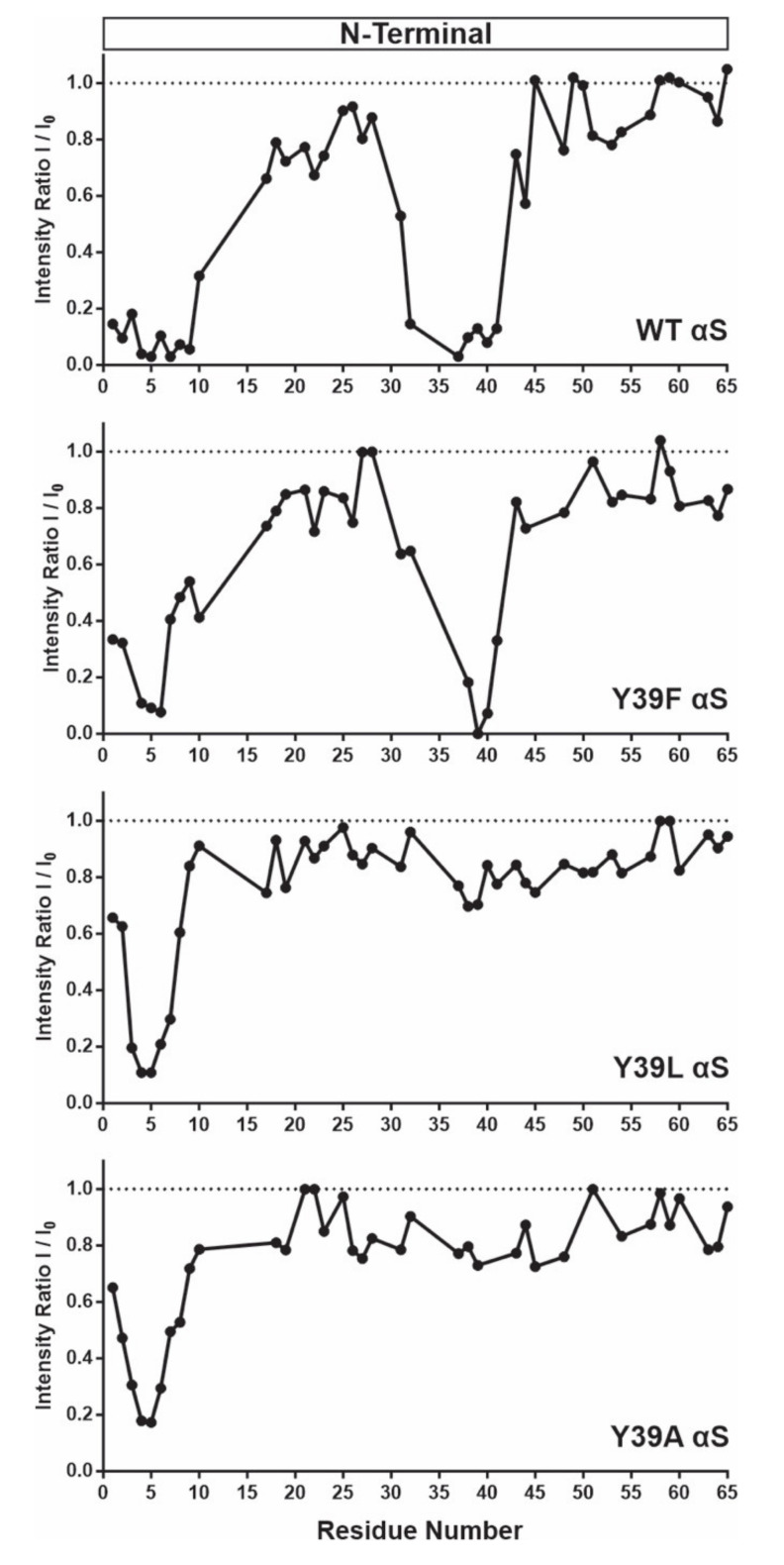
Structural characterization of the interaction between phthalocyanine tetrasulfonate and the Y39 αS mutants monitored by NMR spectroscopy. I/I_0_ profiles of backbone amide groups of 50 µM *wt*, Y39F, Y39L, and Y39A αS proteins in the presence of 50 µM phthalocyanine tetrasulfonate (PcTS). Binding sites for the αS-PcTS complexes at the N-terminal region of the protein are shown. ^1^H–^15^N HSQC spectra were recorded at 15 °C using 15N isotopically enriched αS samples (50 μM) dissolved in Buffer A.

We then studied the mutants Y39F, Y39L, and Y39A of αS, aiming to determine the role of these residues in directing the binding of the anti-amyloid compound PcTS to the N-terminus (Figure 7). As previously shown, complete loss of PcTS binding to the 35–41 region was observed when the Tyr residue in position 39 was replaced by Ala [6]. Replacing the aromatic side-chain by leucine in the Y39L mutant was also sufficient to impair PcTS binding. Conversely, removing the aromatic side chain in position 39 and replacing it with Phe did not alter PcTS interaction, as revealed by the binding features of PcTS at this site. Altogether, these results demonstrate unequivocally that aromaticity at position 39 also had a critical role for PcTS binding to the N-terminus of αS and that specific aromatic interactions with the Y39 residue provided a central mechanistic basis for the inhibitory process of PcTS on αS fibrillation.

## 3. Discussion

In this work, we investigated the importance of aromaticity at position 39 in αS through the analysis of the impact of Y39F, Y39L, and Y39A mutations on the structural properties of its monomeric state and its interaction with the anti-amyloid agent PcTS.

By using biophysical techniques, we showed that removing aromatic functionality at position 39 of monomeric αS does not affect the disordered nature or the secondary structure propensities of the protein. Added to that, our experimental and simulated results indicate that replacement of Tyr-39 by Ala and Leu leads to protein variants that populate more compact conformations. These αS mutants show a modified number and type of contacts compared to *wt* and Y39F species. Assuming more expanded conformations for the αS species containing aromatic residues at position 39 and based on the experimental evidences from previous studies performed on the Y39A protein variant [6,26], one could then predict the following trend of protein fibril assembly propensities: *wt* ~ Y39F > Y39A, Y39L.

On the other hand, contrasting with the subtle changes induced by mutations on the protein structure, removing aromaticity at position 39 not only affects the compactness of the protein but also impacts strongly on the interaction of αS with PcTS. The inhibitory mechanism exerted by phthalocyanines on amyloid assembly is proposed to be a direct consequence of their interaction with target proteins [5]. These molecular interactions are influenced strongly by the highly aromatic character of the cyclic tetrapyrrole ring system, which contributes importantly to the ability of these molecules to bind strongly and selectively to a protein via π−π interactions with aromatic residues. In our previous studies, we showed that the basis for the inhibitory effect of this compound on the amyloid assembly of αS relies on its binding to the Y39 site at the N-terminus sequence of αS [6]. Added to that, in the current study, we performed the structural characterization of the PcTS interaction with both the Y39A and the Y39L variants of the protein, concluding that this interaction is primarily driven by π−π stacking interactions between the aromatic side-chain of Tyr-39 and the aromatic ring system of phthalocyanines. These findings provide clear evidence that aromatic interactions are required for the formation of the αS–PCTS complex, a key event in the molecular pathway that leads to the inhibition of αS fibril formation. 

**Figure 8:**
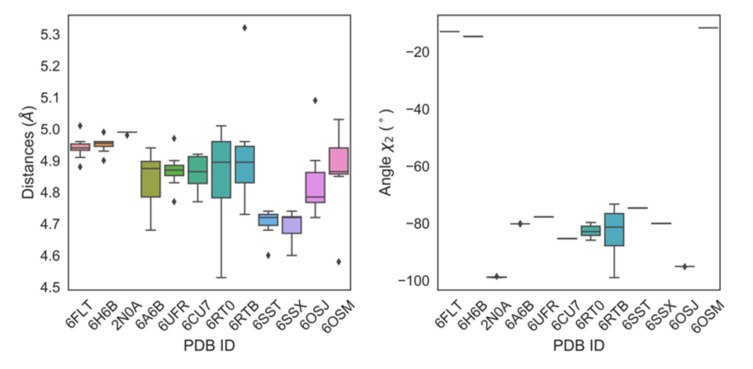
Structural features of fibrillated *wt* αS proteins. Boxplots showing the distribution of values for ring-to-ring distances (left) and χ2 dihedral angles (right) for fibrillated αS proteins in the PDB for Tyr-39.

Indeed, the fact that residue 39 was found to be solvent exposed not only in the monomer but also in transient species formed during the early stages of αS fibrillation [44] implies that Y39 could also be targeted directly by PcTS in early aggregate αS species, a fact that would be consistent with the evidences reporting that PcTS molecules appear mostly incorporated into the αS aggregates. In that direction, the crystal structures of fibrillar αS reveal that Tyr-39 is involved in π−π self-stacking interactions (Figure 8), with ring distances around 4.86 ± 0.12 Å, a very small inter-ring angle, and a χ^2^ dihedral angle of −80 degrees, indicating that Tyr-39 π-stacking might play a role for the aggregation of monomers or oligomers in the earliest stages of αS fibril formation. However, this is not relevant for later stages of the process, such as the dimerization of fibrils, for which the NAC region is known to play a major role [45,46,47]. Indeed, the core region of amyloid fibrils of αS was shown to begin somewhere in the range of residues 31–39 [48,49], precisely in the close vicinity of the primary site targeted by phthalocyanines. 

It is noteworthy that interactions governing the protein–compound reactions seem to be of the same nature of those responsible for protein–protein association reactions. Hence, perturbation of intra- and intermolecular interactions on passing from *wt* αS to the Y39A and the Y39L variants might also affect both the π–π stacking formation and the aggregation process. Our results reported here for the first time open the door toward experimental studies in the interface between molecular biophysics and cellular biology aimed to investigate the relative contributions of π-stacking versus hydrophobic packing in the aggregation of αS.

## 4. Materials and Methods

### 4.1. Proteins and Reagents

Unlabeled and ^15^N isotopically enriched N-terminally acetylated αS and its Y39F, Y39L, and Y39A variants were obtained by co-transforming *Escherichia coli* BL21 with the plasmid harboring the corresponding protein gene and a second one that encodes for the components of yeast NatB acetylase complex [50]. Both plasmids carried different antibiotic resistance, namely ampicillin and chloramphenicol, to select the doubly transformed *E. coli* colonies. Purification was carried out as previously reported [51] with the exception that both antibiotics were included in the growth flasks to avoid plasmid purge during growth and expression. The final purity of the protein samples was determined by SDS-PAGE. Purified protein samples were dissolved in 20 mM 2-(N-morpholino)ethanesulfonic acid (MES) buffer supplemented with 100 mM NaCl at pH 6.5 (Buffer A). Protein concentrations were determined spectrophotometrically by measuring absorption at 274 nm and using an epsilon value of 5600 M^−1·^cm^−1^. 

### 4.2. NMR Experiments

NMR spectra were recorded on a Bruker 600 MHz HD Avance III spectrometer equipped with a cryogenically cooled triple resonance inverse (TCI) ^1^H (^13^C/^15^N) probe. One-dimensional 1D ^1^H-NMR experiments were acquired at 15 °C on 100 µM unlabeled αS samples dissolved in Buffer A. Two-dimensional 2D ^1^H-^15^N heteronuclear single quantum correlation (HSQC) experiments were performed with pulsed-field gradient enhanced pulse sequences on 50 µM ^15^N-labeled protein samples dissolved in Buffer A at 15 °C [52]. Aggregation did not occur under these low temperature conditions and absence of stirring. Mean weighted chemical-shift displacements (MW ^1^H-^15^N ΔCS) were calculated as [(Δδ^1^H)^2^ + (Δδ^15^N)^2^/25]^1/2^ [52]. Three-bond HN-Hα coupling constants (^3^J_HN-Hα_) were obtained from the ratio between the intensities of the diagonal peaks and the cross-peaks in the HNHA experiment, which are three-dimensional experiments designed to accurately determine three-bond H^N^-H^α^ J-coupling constants [53]. Three-bond HN-Hα coupling constants (^3^J_HN-Hα_) are sensitive to the torsion angle φ populated by each residue in the protein sequence and thus report on secondary structure content. This coupling fell in the range 3.0–6.0 Hz for an α-helix and 8.0–11.0 Hz for a β-sheet structure. For a random-coil, a weighted average of these values was observed, which typically ranged between 6.0 and 8.0 Hz for most residues [54,55]. Pulse field gradient-NMR experiments were acquired at 15 °C on 100 µM unlabeled αS samples dissolved in D_2_O and containing dioxane as an internal radius standard (2.12 Å) and viscosity probe. A series of 20 one-dimensional spectra were collected as a function of gradient amplitude. The gradient strength was shifted from 1.69 to 33.72 G cm^−1^ in a linear manner. For the mapping experiments with the PcTS compound, ^1^H-^15^N HSQC amide cross-peaks affected during titrations with the ligand molecule were identified by comparing their intensities (*I*) with those of the same cross-peaks in the data set of free protein (*I*_0_) [6]. The *I*/*I*_0_ ratios of non-overlapping cross-peaks belonging to residues in the N-terminal region were plotted as a function of the protein sequence to obtain the intensity profiles. Acquisition and processing of NMR spectra were performed using TOPSPIN 7.0 (Bruker Biospin, https://www.bruker.com/nc.html Bruker Scientific LLC. Billerica, MA 01821. USA). The 2D spectra analysis and visualization were performed with CCPN (Collaborative Computational Project for NMR).

### 4.3. CD Spectroscopy

αS samples were diluted 10-fold in Buffer A, and CD spectra were recorded on a JASCO J-530 spectropolarimeter (JASCO, https://jascoinc.com, Mary’s Court Easton, MD 21601. USA).

### 4.4. Molecular Simulations

The calculations were based on our predicted conformational ensemble of *wt* monomeric, human N-terminus acetylated alpha synuclein [56]. Our prediction was shown to be fully consistent with available experimental data [56]. We first clustered the conformational ensemble according to the *gromos* algorithm proposed by Daura et al. [57] and implemented in the software GROMACS 2016.4 (http://www.gromacs.org) [58]. Then, we selected the most extended structure among the cluster representatives. The Y39F, the Y39L, and the Y39A mutations were created using the Schrödinger software [59]. The *wt* and the three variants were solvated in a truncated dodecahedral box insuring at least 1.0 nm of solvation shell and neutralized with Na^+^ and Cl^−^ and ions until achieving an excess salt concentration of 15 mM. The topology and the coordinate files were prepared using GROMACS 2016.4 patched with the software PLUMED 2.4.0 (https://www.plumed.org) [60]. 

The force field for the water, the counterions, and the protein was a99SB-disp [61]. Simulations based on the latter have been shown to reproduce a variety of structural properties of non-folded proteins [61]. The Particle Mesh Ewald method [62] was used to treat the long-range interactions with a real space cutoff of 12 Å. The same cutoff was used for the van der Waals interactions. The LINear Constraint Solver (LINCS) algorithm [63] was used to constrain all bond lengths. Constant pressure and temperature conditions were considered coupling the systems to a Nosé–Hoover thermostat [64,65] at 288 K and a Parrinello–Rahman barostat [66] at 1 atm. A timestep of 2 fs was used for the simulations.

After 50 ns of plain MD simulations, the *wt* and the three variants underwent 25 ns of Hamiltonian replica exchange with solute tempering (REST2) simulations [30]. The same computational setup as above was applied. For an adequate sampling, 26 replicas were distributed in a range from 288 K to 533 K, achieving an exchange probability around 0.25 during the simulation. The range of temperatures was chosen for promoting the conformational sampling without increasing significantly the number of residues outside the allowed Ramachandran areas, similar to previous works [56]. 

A variety of properties were calculated for the last 20 ns of the replica with the lowest temperature, either using the full ensemble of structures (items (i)–(iv)) or five representative conformations spanning ca. 65% of the conformational space, identified with a cluster analysis using the *gmx cluster* and the *gromos* algorithm [57] in GROMACS 2016.4 (items (v)–(vii)): (i) the radius of gyration (RG) and end-to end distance (EE) with the *gmx polystat* code from GROMACS 2016.4 [58]; (ii) the inter-residue contacts with the *gmx select* code from GROMACS 2016.4; (iii) the H-bonds (HB) and the salt-bridges (SB) with the *gmx hbond* code from GROMACS 2016.4 or VMD [67]. A hydrogen bond was considered to be formed when acceptor and donor heavy atoms were at distance of 3.5 Å or lower, and the angle formed by acceptor, hydrogen atoms, and donor was of 30 degrees or less, while, for a salt bridge, the distance between the nitrogen atoms of basic residues and the oxygen atoms of acid residues was set to no more than 3.2 Å; (iv) the hydrophobic contacts analysis, the distance between heavy atom side-chains, was computed with *gmx distance* and *gmx mdmat* codes present in GROMACS 2016.4. The following residues were considered for the analysis: Ala, Leu, Met, Val, Phe, Pro, Tyr, and Ile. A cut-off of 6.0 Å was set. We considered contacts occurring for 15% or more of the simulation time. Analysis with different choices of these conditions turned out not to differ from our (qualitative) conclusions regarding the hydrophobic interactions in Section 2.2 (data not shown); (v) the circular dichroism (CD) spectrum with PDB2CD (https://pdb2cd.cryst.bbk.ac.uk) [68]; (vi) the chemical shifts of the C, Cα, Cβ_,_ and N atoms, with SHIFTX2 1.10 (http://www.shiftx2.ca) [69]; (vii) the secondary structure elements with DSSP (https://swift.cmbi.umcn.nl/gv/dssp/) [70]. 

## Figures and Tables

**Table 1:** Number of residues forming different secondary structure elements, as predicted by our REST2 simulation. The averages values and the corresponding standard deviations are reported.

Secondary Structure	*wt*	Y39F	Y39L	Y39A
**Coil**	72 ± 6	67 ± 6	59 ± 6	68 ± 6
**Bend**	30 ± 5	31 ± 4	32 ± 5	25 ± 5
**Turn**	19 ± 5	23 ± 6	25 ± 5	24 ± 6
**α-Helix**	11 ± 4	8 ± 5	11 ± 4	11 ± 4
**3-Helix**	5 ± 4	7 ± 4	7 ± 4	6 ± 4
**β-Sheet**	2 ± 4	1 ± 2	3 ± 3	2 ± 2
**β-Bridge**	1 ± 2	3 ± 2	4 ± 2	4 ± 2

## Supplementary Materials

Supplementary materials can be found at https://www.mdpi.com/1422-0067/21/14/5061/s1. Figure S1. Overlaid contour plots of ^1^H-^15^N HSQC spectra of 50 μM *wt* (black) and Y39L αS (red), showing all amide resonances detected. Most-affected residues located in the vicinity of the mutated site are labeled. Figure S2. Secondary structure content for individual residues across the four species investigated here during the simulation. Table S1. Characteristics of the RG and EE distributions across the four variants. The centers depicted in Figure 6 are described as the respective joint distributions. Additionally, the values of the mode and average of the marginal distributions (the univariate, independent ones) are depicted below. The conclusions of the study were based on the joint distribution of EE and RG. Table S2. Intramolecular HB content for the four variants within the structural ensemble. Table S3. Intramolecular hydrophobic contacts for the four variants within the structural ensemble.
