# Supplementary material for: Role of Tyr-39 for the Structural Features of α-Synuclein and for the Interaction with a Strong Modulator of Its Amyloid Assembly
Source: Int J Mol Sci. 2020 Jul 17;21(14):5061. doi: 10.3390/ijms21145061 (PMC7404028; doi:10.3390/ijms21145061)
Supplement: Supplementary file 1 [file ijms-21-05061-s001.pdf]

International Journal of
Molecular Sciences

MDPI

Supplementary information

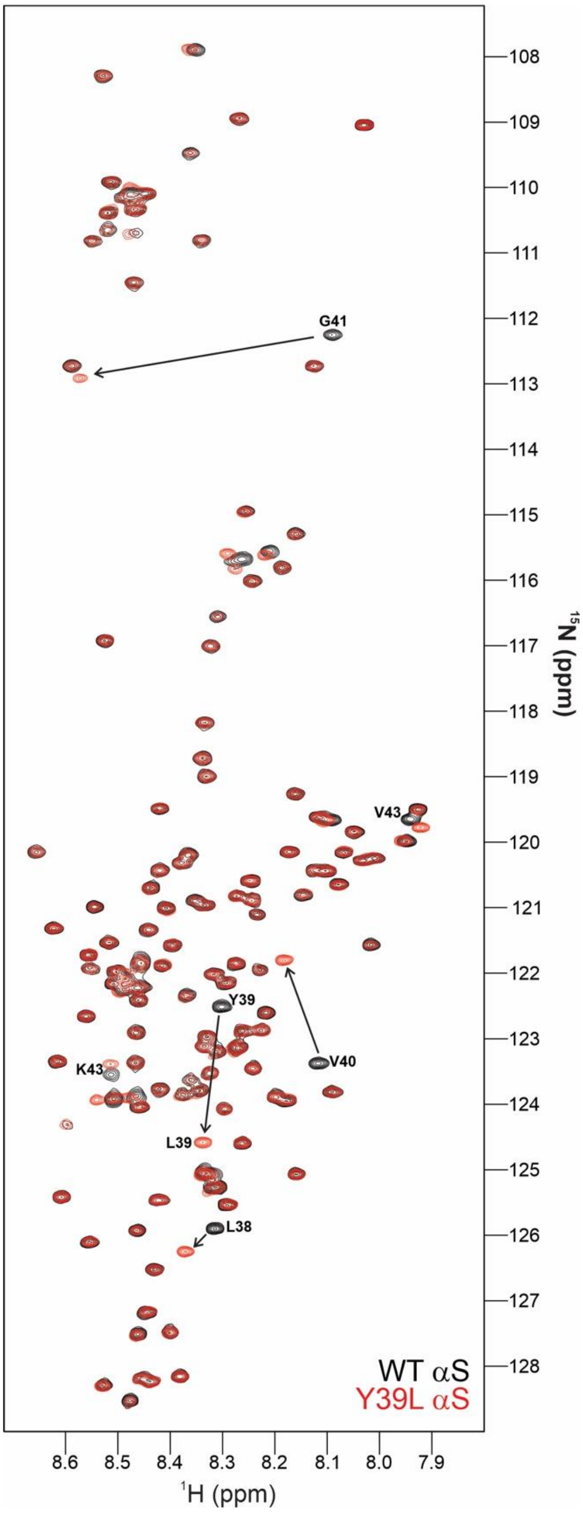

Figure S1. Overlaid contour plots of ^{1}H-^{15}N HSQC spectra of 50 μM *wt* (black) and Y39L αS (red), showing all amide resonances detected. Most-affected residues located in the vicinity of the mutated site are labeled.

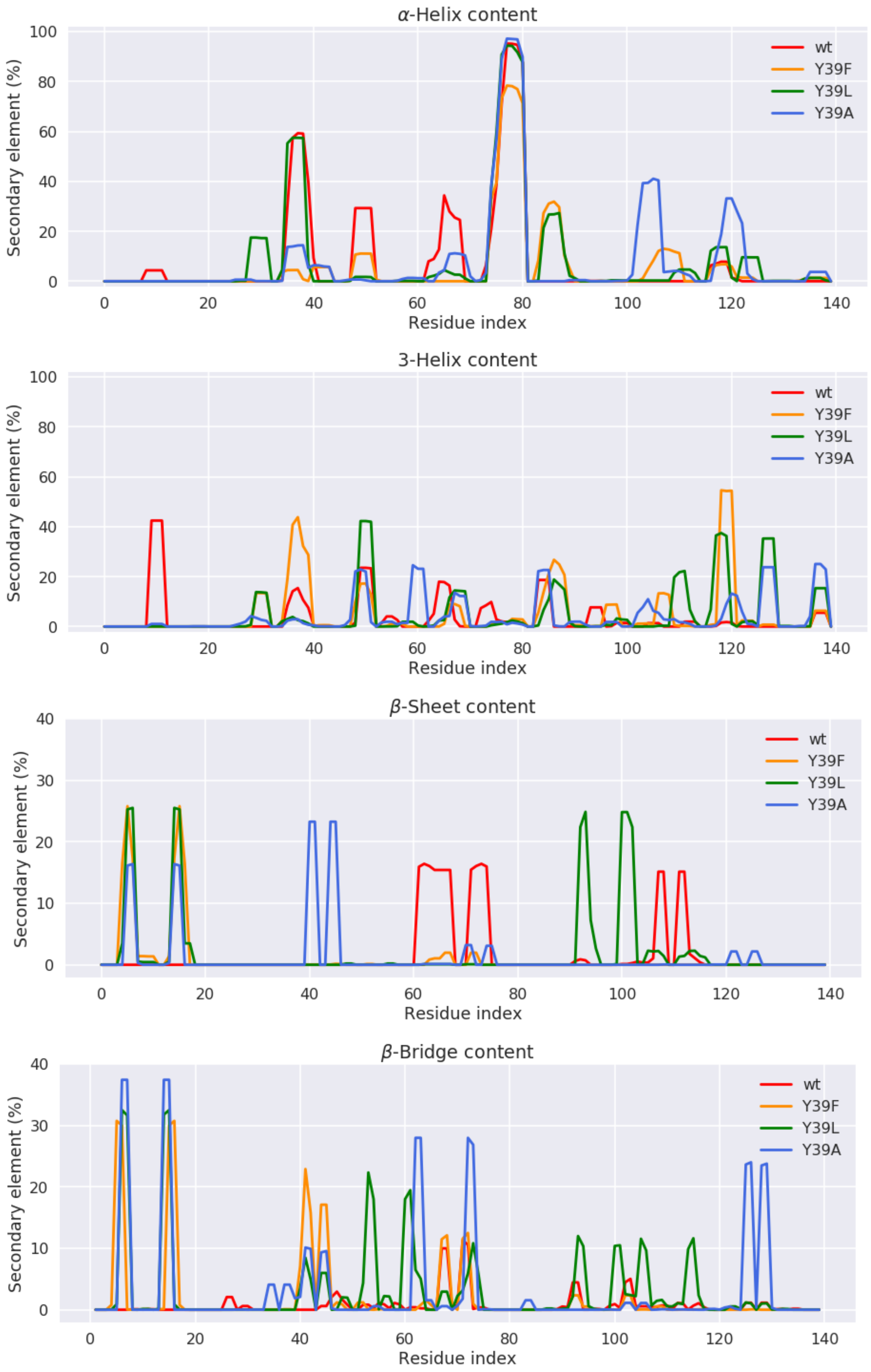

Figure S2. Secondary structure content for individual residues across the four species investigated here during the simulation.

Table S1. Characteristics of the RG and EE distributions across the four variants. The centers depicted in Figure 6 are described as the respective joint distributions. Additionally, the values of the mode and average of the marginal distributions (the univariate, independent ones) are depicted below. The conclusions of the study were based on the joint distribution of EE and RG.

	**Observable**	***wt***	**Y39F**	**Y39L**	**Y39A**
Joint distribution	EE	11.7	10.6	8.3	6.0
	RG	4.1	3.6	3.3	2.7
Marginal distribution	Average (EE)	9.7 ± 2.1	9.0 ± 2.0	9.4 ± 1.6	8.7 ± 2.5
	Average (RG)	3.4 ± 0.6	3.4 ± 0.4	3.6 ± 0.5	3.1 ± 0.3
	Mode (EE)	9.6	10.2	8.5	10.8
	Mode (RG)	4.2	3.3	3.4	2.8

Table S2. Intramolecular HB content for the four variants within the structural ensemble.

***wt*:**

**Donor**	**Acceptor**	**Donor Region**	**Acceptor Region**	**Intra-Region**	**HB Percentage**
PHE4-Main	MET1-Main	N-term	N-term	Yes	79.3
MET5-Main	MET1-Main	N-term	N-term	Yes	74.6
VAL63-Main	THR59-Main	NAC	N-term	No	68.8
THR64-Side	LYS60-Main	NAC	N-term	No	67.2
THR64-Main	LYS60-Main	NAC	N-term	No	66.9
GLU131-Main	PRO128-Main	C-term	C-term	Yes	47.5
LYS58-Main	THR54-Main	N-term	N-term	Yes	45.1
THR59-Main	GLN62-Side	N-term	NAC	No	41.1
GLU57-Main	THR54-Main	N-term	N-term	Yes	40.4
THR72-Main	VAL70-Main	NAC	NAC	Yes	39.0
GLY73-Main	ALA69-Main	NAC	NAC	Yes	38.7
GLN62-Main	THR59-Side	NAC	N-term	No	36.7
GLU105-Main	GLY101-Main	C-term	C-term	Yes	35.4
ASN65-Main	GLU61-Main	NAC	NAC	Yes	33.7
VAL77-Main	VAL74-Main	NAC	NAC	Yes	32.9
GLU104-Main	LEU100-Main	C-term	C-term	Yes	32.8
ASP2-Main	SER9-Side	N-term	N-term	Yes	32.7
ALA91-Main	ILE88-Main	NAC	NAC	Yes	30.9
LYS96-Side	GLU104-Side	C-term	C-term	Yes	29.2
GLY132-Main	SER129-Main	C-term	C-term	Yes	28.3
GLY93-Main	ALA89-Main	NAC	NAC	Yes	27.5
ALA107-Main	LYS102-Main	C-term	C-term	Yes	26.0
GLY132-Main	PRO128-Main	C-term	C-term	Yes	24.2
ALA76-Main	GLY73-Main	NAC	NAC	Yes	24.1
THR92-Main	ILE88-Main	NAC	NAC	Yes	24.1
VAL49-Main	VAL37-Main	N-term	N-term	Yes	23.9
MET1-Main	PHE4-Side	N-term	N-term	Yes	23.3
VAL66-Main	GLN62-Main	NAC	NAC	Yes	22.8
ALA124-Main	ASN122-Side	C-term	C-term	Yes	22.1
ALA69-Main	GLY73-Main	NAC	NAC	Yes	21.6
GLY106-Main	LYS102-Main	C-term	C-term	Yes	21.3
THR75-Main	VAL71-Main	NAC	NAC	Yes	21.3
GLY36-Main	GLN24-Main	N-term	N-term	Yes	20.8
GLY41-Main	VAL37-Main	N-term	N-term	Yes	20.0

VAL82-Main	GLN79-Main	NAC	NAC	Yes	18.4
LYS58-Main	VAL55-Main	N-term	N-term	Yes	18.1
THR92-Main	ALA89-Main	NAC	NAC	Yes	17.8
TYR133-Main	PRO128-Main	C-term	C-term	Yes	17.3
SER9-Side	ASP2-Side	N-term	N-term	Yes	16.2
ALA107-Main	GLY101-Main	C-term	C-term	Yes	16.0
GLY41-Main	GLY47-Main	N-term	N-term	Yes	14.7
GLY106-Main	ASN103-Main	C-term	C-term	Yes	14.1
LYS34-Main	ALA27-Main	N-term	N-term	Yes	14.0
ALA78-Main	GLY67-Main	NAC	NAC	Yes	13.8
ALA69-Main	VAL66-Main	NAC	NAC	Yes	13.7
THR44-Side	GLU46-Main	N-term	N-term	Yes	13.6
GLY67-Main	ALA78-Main	NAC	NAC	Yes	13.5
LYS80-Main	ASN65-Main	NAC	NAC	Yes	13.5
LYS96-Side	ASP98-Side	C-term	C-term	Yes	13.4
ASN65-Side	VAL82-Main	NAC	NAC	Yes	13.1
ALA29-Main	LYS32-Main	N-term	N-term	Yes	12.1
GLY67-Main	THR64-Main	NAC	NAC	Yes	11.9
GLN134-Side	GLY132-Main	C-term	C-term	Yes	11.6
ASN65-Main	GLN62-Main	NAC	NAC	Yes	11.5
TYR39-Main	GLY47-Main	N-term	N-term	Yes	11.4
ALA76-Main	THR72-Main	NAC	NAC	Yes	11.3
GLU105-Main	LYS102-Main	C-term	C-term	Yes	11.1
LYS12-Side	ASP2-Side	N-term	N-term	Yes	11.1
THR54-Main	GLU57-Side	N-term	N-term	Yes	10.4
GLU104-Main	GLY101-Main	C-term	C-term	Yes	10.4
ASN103-Main	LEU100-Main	C-term	C-term	Yes	10.0
GLY68-Main	ASN65-Main	NAC	NAC	Yes	9.8
LYS32-Main	ALA29-Main	N-term	N-term	Yes	9.7
LYS102-Side	GLU105-Side	C-term	C-term	Yes	9.6
GLN109-Side	GLY111-Main	C-term	C-term	Yes	9.5
VAL66-Main	VAL63-Main	NAC	NAC	Yes	9.3
VAL63-Side	THR59-Main	NAC	N-term	No	9.3
ASN103-Side	ALA107-Main	C-term	C-term	Yes	9.1
GLY14-Main	LYS12-Main	N-term	N-term	Yes	8.9
GLN62-Main	THR59-Main	NAC	N-term	No	8.6
GLY67-Main	VAL63-Main	NAC	NAC	Yes	8.4
VAL40-Main	VAL37-Main	N-term	N-term	Yes	8.4
ASN103-Main	GLN99-Main	C-term	C-term	Yes	8.3
THR75-Side	THR72-Main	NAC	NAC	Yes	8.1

Y39F:

Donor	Acceptor	Donor Region	Acceptor Region	Intra-Region	HB Percentage
ASP135-Main	TYR125-Main	C-term	C-term	Yes	85.9
TYR125-Main	ASP135-Main	C-term	C-term	Yes	81.3
MET5-Main	MET1-Main	N-term	N-term	Yes	75.9
PHE4-Main	MET1-Main	N-term	N-term	Yes	61.9
VAL63-Main	THR59-Main	NAC	N-term	No	60.8

THR64-Side	LYS60-Main	NAC	N-term	No	58.5
ASN122-Main	ASP119-Main	C-term	C-term	Yes	53.7
THR22-Main	ALA19-Main	N-term	N-term	Yes	53.3
THR59-Main	GLN62-Side	N-term	NAC	No	49.2
ALA124-Main	ASN122-Side	C-term	C-term	Yes	48.4
ASN65-Main	GLU61-Main	NAC	NAC	Yes	47.4
VAL49-Main	VAL37-Main	N-term	N-term	Yes	47.2
GLY73-Main	ALA69-Main	NAC	NAC	Yes	47.1
THR22-Side	ALA19-Main	N-term	N-term	Yes	45.2
LYS23-Main	GLU20-Main	N-term	N-term	Yes	43.5
GLN62-Main	THR59-Side	NAC	N-term	No	43.3
THR64-Main	LYS60-Main	NAC	N-term	No	42.3
THR54-Main	GLY51-Main	N-term	N-term	Yes	39.8
THR72-Main	VAL70-Main	NAC	NAC	Yes	39.6
VAL40-Main	GLY47-Main	N-term	N-term	Yes	39.2
ALA76-Main	GLY73-Main	NAC	NAC	Yes	39.2
GLU105-Main	LYS102-Main	C-term	C-term	Yes	36.1
GLY67-Main	VAL63-Main	NAC	NAC	Yes	34.4
THR54-Side	GLY51-Main	N-term	N-term	Yes	34.1
ALA18-Main	VAL15-Main	N-term	N-term	Yes	33.6
GLY14-Main	LYS12-Main	N-term	N-term	Yes	30.0
GLU104-Main	GLY101-Main	C-term	C-term	Yes	27.2
ALA91-Main	ILE88-Main	NAC	NAC	Yes	27.0
GLN24-Main	GLU13-Main	N-term	N-term	Yes	25.6
SER9-Side	ALA11-Main	N-term	N-term	Yes	25.2
GLY41-Main	GLY47-Main	N-term	N-term	Yes	24.1
GLU35-Main	LYS32-Main	N-term	N-term	Yes	24.0
ASP121-Main	ASP119-Side	C-term	C-term	Yes	22.0
GLU57-Main	ALA53-Main	N-term	N-term	Yes	22.0
VAL66-Main	GLN62-Main	NAC	NAC	Yes	21.8
TYR125-Side	GLU137-Side	C-term	C-term	Yes	21.0
ASN122-Side	TYR136-Side	C-term	C-term	Yes	20.4
VAL55-Main	VAL52-Main	N-term	N-term	Yes	20.0
LYS102-Side	GLU105-Side	C-term	C-term	Yes	19.9
GLY68-Main	VAL74-Main	NAC	NAC	Yes	19.4
THR44-Side	GLU46-Main	N-term	N-term	Yes	18.9
GLU130-Main	MET127-Main	C-term	C-term	Yes	17.3
GLN99-Main	LYS96-Main	C-term	C-term	Yes	17.1
LYS97-Side	ASP98-Side	C-term	C-term	Yes	16.7
GLY93-Main	ALA89-Main	NAC	NAC	Yes	16.4
THR44-Main	GLY41-Main	N-term	N-term	Yes	16.0
GLU46-Main	THR44-Side	N-term	N-term	Yes	15.9
LYS23-Main	ALA19-Main	N-term	N-term	Yes	15.8
GLY106-Main	ASN103-Main	C-term	C-term	Yes	15.7
GLU57-Main	THR54-Main	N-term	N-term	Yes	15.3
THR92-Main	ALA89-Main	NAC	NAC	Yes	15.1
ALA56-Main	VAL52-Main	N-term	N-term	Yes	14.9
THR44-Side	VAL40-Main	N-term	N-term	Yes	14.2
ASP2-Main	ALA11-Main	N-term	N-term	Yes	13.5
ALA69-Main	GLY73-Main	NAC	NAC	Yes	13.5
LYS58-Main	VAL55-Main	N-term	N-term	Yes	13.2
THR54-Side	HIS50-Main	N-term	N-term	Yes	12.9

VAL66-Main	VAL63-Main	NAC	NAC	Yes	12.6
LYS96-Main	GLN99-Main	C-term	C-term	Yes	12.6
THR33-Main	ALA29-Main	N-term	N-term	Yes	12.2
THR54-Main	HIS50-Main	N-term	N-term	Yes	12.1
TYR136-Main	ASP135-Side	C-term	C-term	Yes	11.8
THR22-Main	ALA18-Main	N-term	N-term	Yes	11.8
LYS58-Main	THR54-Main	N-term	N-term	Yes	11.7
THR33-Side	ALA29-Main	N-term	N-term	Yes	11.5
GLU130-Main	PRO128-Main	C-term	C-term	Yes	11.5
THR92-Main	ILE88-Main	NAC	NAC	Yes	11.4
ASP98-Main	LYS96-Main	C-term	C-term	Yes	11.2
GLN62-Side	THR59-Side	NAC	N-term	No	11.0
GLY93-Main	ALA90-Main	NAC	NAC	Yes	10.7
ASN122-Side	GLN134-Side	C-term	C-term	Yes	10.4
ALA78-Main	VAL63-Main	NAC	NAC	Yes	10.3
VAL63-Side	THR59-Main	NAC	N-term	No	10.1
GLN134-Side	TYR133-Side	C-term	C-term	Yes	10.0
GLN62-Main	THR59-Main	NAC	N-term	No	9.9
ASN103-Main	LEU100-Main	C-term	C-term	Yes	9.5
MET127-Main	TYR133-Main	C-term	C-term	Yes	9.5
LYS97-Side	GLU104-Side	C-term	C-term	Yes	9.2
LYS34-Main	GLY31-Main	N-term	N-term	Yes	9.2
HIS50-Main	VAL37-Main	N-term	N-term	Yes	9.0
LYS32-Side	GLU28-Side	N-term	N-term	Yes	8.9
PHE39-Main	GLY47-Main	N-term	N-term	Yes	8.8
LYS34-Main	ALA30-Main	N-term	N-term	Yes	8.7
TYR125-Side	ASP119-Main	C-term	C-term	Yes	8.6
LYS32-Side	GLU35-Side	N-term	N-term	Yes	8.5
GLU35-Main	GLY31-Main	N-term	N-term	Yes	8.5
ALA56-Main	ALA53-Main	N-term	N-term	Yes	8.2

Y39L:

Donor	Acceptor	Donor Region	Acceptor Region	Intra-Region	HB Percentage
ASP135-Main	TYR125-Main	C-term	C-term	Yes	86.7
MET127-Main	TYR133-Main	C-term	C-term	Yes	85.1
VAL63-Main	THR59-Main	NAC	N-term	No	73.4
GLU105-Main	GLY101-Main	C-term	C-term	Yes	69.1
THR59-Main	GLN62-Side	N-term	NAC	No	68.8
PHE4-Main	MET1-Main	N-term	N-term	Yes	63.1
THR64-Side	LYS60-Main	NAC	N-term	No	59.4
GLN62-Main	THR59-Side	NAC	N-term	No	56.3
VAL26-Main	GLU35-Main	N-term	N-term	Yes	56.1
MET5-Main	MET1-Main	N-term	N-term	Yes	56.1
ASN65-Main	GLU61-Main	NAC	NAC	Yes	54.3
THR64-Main	LYS60-Main	NAC	N-term	No	54.0
ALA107-Main	LYS102-Main	C-term	C-term	Yes	53.1
ILE88-Main	LYS80-Main	NAC	NAC	Yes	42.8
VAL77-Main	GLY73-Main	NAC	NAC	Yes	42.7
LYS23-Main	GLU20-Main	N-term	N-term	Yes	42.1

ALA91-Main	ILE88-Main	NAC	NAC	Yes	40.5
LYS97-Side	GLU105-Side	C-term	C-term	Yes	40.5
GLY73-Main	VAL70-Main	NAC	NAC	Yes	39.1
THR54-Main	GLY51-Main	N-term	N-term	Yes	36.6
VAL49-Main	VAL37-Main	N-term	N-term	Yes	35.8
ALA76-Main	GLY73-Main	NAC	NAC	Yes	35.0
VAL40-Main	GLY47-Main	N-term	N-term	Yes	33.8
SER87-Side	VAL82-Main	NAC	NAC	Yes	32.5
GLY67-Main	VAL63-Main	NAC	NAC	Yes	30.6
LYS97-Side	ASP98-Side	C-term	C-term	Yes	29.6
VAL118-Main	ASP115-Main	C-term	C-term	Yes	28.8
VAL66-Main	GLN62-Main	NAC	NAC	Yes	27.6
VAL15-Main	LYS12-Main	N-term	N-term	Yes	27.6
LYS32-Main	ALA29-Main	N-term	N-term	Yes	26.6
GLN24-Main	LYS21-Main	N-term	N-term	Yes	26.6
ALA69-Main	VAL77-Main	NAC	NAC	Yes	25.4
LEU113-Main	GLN109-Main	C-term	C-term	Yes	24.9
SER9-Side	VAL15-Main	N-term	N-term	Yes	24.6
GLN79-Main	VAL66-Main	NAC	NAC	Yes	24.2
THR54-Side	GLY51-Main	N-term	N-term	Yes	23.5
THR92-Main	ALA89-Main	NAC	NAC	Yes	23.4
ALA56-Main	VAL52-Main	N-term	N-term	Yes	20.9
VAL55-Main	GLY51-Main	N-term	N-term	Yes	20.5
ALA11-Main	SER9-Side	N-term	N-term	Yes	19.9
GLY111-Main	PRO108-Main	C-term	C-term	Yes	19.2
LYS80-Main	GLY86-Main	NAC	NAC	Yes	18.9
ASP2-Main	SER9-Side	N-term	N-term	Yes	18.5
GLU130-Main	PRO128-Main	C-term	C-term	Yes	18.4
THR33-Side	GLN24-Main	N-term	N-term	Yes	18.2
LYS32-Main	ALA27-Main	N-term	N-term	Yes	18.0
LYS34-Main	VAL26-Main	N-term	N-term	Yes	17.9
ALA76-Main	THR72-Main	NAC	NAC	Yes	17.4
VAL55-Main	VAL52-Main	N-term	N-term	Yes	17.2
THR81-Main	ASN65-Main	NAC	NAC	Yes	16.1
GLY47-Main	VAL40-Main	N-term	N-term	Yes	15.9
VAL82-Main	ASN65-Main	NAC	NAC	Yes	15.7
ILE112-Main	GLN109-Main	C-term	C-term	Yes	15.4
GLU104-Main	GLY101-Main	C-term	C-term	Yes	14.5
GLN79-Side	GLY68-Main	NAC	NAC	Yes	14.5
THR44-Side	GLU46-Side	N-term	N-term	Yes	14.3
GLU35-Main	VAL26-Main	N-term	N-term	Yes	13.5
GLU46-Main	VAL40-Main	N-term	N-term	Yes	13.4
THR75-Main	THR72-Main	NAC	NAC	Yes	13.3
MET5-Main	ASP2-Main	N-term	N-term	Yes	12.8
GLU130-Main	MET127-Main	C-term	C-term	Yes	12.8
GLU57-Main	ALA53-Main	N-term	N-term	Yes	12.7
HIS50-Main	SER42-Side	N-term	N-term	Yes	12.5
SER87-Side	GLY84-Main	NAC	NAC	Yes	12.4
LYS96-Side	GLU104-Side	C-term	C-term	Yes	12.4
ILE112-Main	PRO108-Main	C-term	C-term	Yes	12.3
THR92-Main	ILE88-Main	NAC	NAC	Yes	12.2
GLY106-Main	ASN103-Main	C-term	C-term	Yes	12.1

GLN134-Side	ASP121-Main	C-term	C-term	Yes	12.1
ASP2-Main	VAL16-Main	N-term	N-term	Yes	12.1
SER42-Main	LEU39-Main	N-term	N-term	Yes	11.9
VAL66-Main	VAL63-Main	NAC	NAC	Yes	11.8
LYS102-Side	GLY111-Main	C-term	C-term	Yes	11.5
LYS23-Side	GLU20-Side	N-term	N-term	Yes	11.4
GLN134-Side	ALA124-Main	C-term	C-term	Yes	11.4
VAL63-Side	THR59-Main	NAC	N-term	No	11.1
SER87-Main	GLY84-Main	NAC	NAC	Yes	10.9
VAL74-Main	VAL71-Main	NAC	NAC	Yes	10.8
GLY67-Main	THR64-Main	NAC	NAC	Yes	10.8
TYR136-Main	ASP135-Side	C-term	C-term	Yes	10.6
ALA89-Main	VAL66-Main	NAC	NAC	Yes	10.6
GLY106-Main	LYS102-Main	C-term	C-term	Yes	10.4
LEU39-Main	GLY47-Main	N-term	N-term	Yes	10.2
GLU83-Main	ASN65-Main	NAC	NAC	Yes	10.1
LYS32-Side	GLU28-Side	N-term	N-term	Yes	10.1
MET1-Main	PHE4-Side	N-term	N-term	Yes	10.0
LYS12-Side	GLU57-Side	N-term	N-term	Yes	9.9
GLY25-Main	LYS21-Main	N-term	N-term	Yes	9.2
GLY47-Main	THR44-Main	N-term	N-term	Yes	9.1
THR92-Side	ILE88-Main	NAC	NAC	Yes	9.0
LYS80-Side	ALA85-Main	NAC	NAC	Yes	9.0
GLY31-Main	GLU28-Main	N-term	N-term	Yes	8.9
GLN24-Side	ALA19-Main	N-term	N-term	Yes	8.8
ASN103-Side	GLU104-Side	C-term	C-term	Yes	8.8
GLN24-Main	GLU20-Main	N-term	N-term	Yes	8.5
GLY14-Main	ALA11-Main	N-term	N-term	Yes	8.3
ALA56-Main	ALA53-Main	N-term	N-term	Yes	8.1

Y39A:

Donor	Acceptor	Donor Region	Acceptor Region	Intra-Region	HB Percentage
MET127-Main	TYR133-Main	C-term	C-term	Yes	91.1
ASP135-Main	TYR125-Main	C-term	C-term	Yes	89.8
GLY132-Main	PRO128-Main	C-term	C-term	Yes	86.7
THR64-Main	LYS60-Main	NAC	N-term	No	75.7
GLU131-Main	PRO128-Main	C-term	C-term	Yes	73.3
VAL77-Main	GLY73-Main	NAC	NAC	Yes	64.2
THR64-Side	LYS60-Main	NAC	N-term	No	63.3
VAL63-Main	THR59-Main	NAC	N-term	No	61.7
GLY73-Main	VAL70-Main	NAC	NAC	Yes	60.2
GLY93-Main	ALA89-Main	NAC	NAC	Yes	53.0
GLN79-Main	GLY67-Main	NAC	NAC	Yes	52.7
ALA76-Main	GLY73-Main	NAC	NAC	Yes	52.7
VAL15-Main	LYS12-Main	N-term	N-term	Yes	50.0

THR22-Main	ALA18-Main	N-term	N-term	Yes	48.0
LYS58-Main	THR54-Main	N-term	N-term	Yes	47.0
THR81-Main	ALA78-Main	NAC	NAC	Yes	45.2
ASN65-Main	GLU61-Main	NAC	NAC	Yes	43.3
GLU57-Main	THR54-Main	N-term	N-term	Yes	42.6
LYS12-Main	VAL15-Main	N-term	N-term	Yes	41.5
ALA69-Main	VAL77-Main	NAC	NAC	Yes	41.4
THR92-Main	ALA89-Main	NAC	NAC	Yes	39.8
THR59-Main	GLN62-Side	N-term	NAC	No	37.0
LEU38-Main	LYS34-Main	N-term	N-term	Yes	34.7
GLY67-Main	VAL63-Main	NAC	NAC	Yes	31.7
GLY68-Main	VAL63-Main	NAC	NAC	Yes	31.6
GLN62-Main	THR59-Side	NAC	N-term	No	30.3
LYS96-Main	GLN99-Main	C-term	C-term	Yes	30.2
VAL82-Main	ALA78-Main	NAC	NAC	Yes	28.5
ALA78-Main	THR81-Side	NAC	NAC	Yes	27.6
VAL37-Main	THR33-Main	N-term	N-term	Yes	27.6
VAL66-Main	GLN62-Main	NAC	NAC	Yes	26.6
PHE4-Main	MET1-Main	N-term	N-term	Yes	25.7
GLN99-Main	LYS96-Main	C-term	C-term	Yes	24.9
LYS21-Main	ALA18-Main	N-term	N-term	Yes	22.8
GLU35-Main	THR33-Side	N-term	N-term	Yes	21.7
GLY14-Main	ALA11-Main	N-term	N-term	Yes	19.8
LYS21-Main	ALA17-Main	N-term	N-term	Yes	19.4
THR22-Side	ALA18-Main	N-term	N-term	Yes	19.1
GLY106-Main	ASN103-Main	C-term	C-term	Yes	18.6
GLU105-Main	GLY101-Main	C-term	C-term	Yes	18.4
TYR136-Main	ASP135-Side	C-term	C-term	Yes	18.1
THR81-Side	ALA78-Main	NAC	NAC	Yes	18.1
MET5-Main	ASP2-Main	N-term	N-term	Yes	17.8
GLN79-Side	GLY68-Main	NAC	NAC	Yes	17.4
GLY67-Main	THR64-Main	NAC	NAC	Yes	16.0
GLU105-Main	ASN103-Side	C-term	C-term	Yes	15.7
VAL82-Main	GLN79-Main	NAC	NAC	Yes	14.6
GLY101-Main	PHE94-Main	C-term	NAC	No	14.5
ALA39-Main	GLY36-Main	N-term	N-term	Yes	14.0
LYS58-Main	VAL55-Main	N-term	N-term	Yes	13.6
VAL74-Main	VAL70-Main	NAC	NAC	Yes	13.1
GLU104-Main	GLY101-Main	C-term	C-term	Yes	12.7
GLY51-Main	MET5-Main	N-term	N-term	Yes	12.5
VAL63-Side	THR59-Main	NAC	N-term	No	11.9
VAL66-Main	VAL63-Main	NAC	NAC	Yes	11.8
THR92-Side	ALA89-Main	NAC	NAC	Yes	11.7
GLY93-Main	ALA90-Main	NAC	NAC	Yes	11.4
THR44-Main	SER42-Side	N-term	N-term	Yes	11.3
GLN62-Main	THR59-Main	NAC	N-term	No	10.5

GLY36-Main	THR33-Side	N-term	N-term	Yes	10.4
LEU100-Main	GLU104-Side	C-term	C-term	Yes	10.3
GLY36-Main	THR33-Main	N-term	N-term	Yes	10.1
LYS23-Main	ALA19-Main	N-term	N-term	Yes	10.1
LYS58-Side	VAL52-Main	N-term	N-term	Yes	9.9
VAL40-Main	GLU35-Main	N-term	N-term	Yes	9.6
GLN62-Side	THR59-Side	NAC	N-term	No	8.4
LYS23-Main	GLU20-Main	N-term	N-term	Yes	8.3

Table S3. Intramolecular hydrophobic contacts for the four variants within the structural ensemble.

Y39	F39	L39	A39	R1	R2	Region1	Region2	Intra-Region
99.8	99.8	99.8	99.8	0ACE	MET1	N-term	N-term	Yes
1.0	0.8	1.3	12.6	0ACE	VAL3	N-term	N-term	Yes
76.6	58.0	69.7	29.5	0ACE	PHE4	N-term	N-term	Yes
94.4	90.3	85.7	13.3	0ACE	MET5	N-term	N-term	Yes
72.1	83.9	66.0	0.1	0ACE	ALA11	N-term	N-term	Yes
2.6	4.8	0.1	0.3	0ACE	VAL15	N-term	N-term	Yes
7.9	0.5	2.9	0.6	0ACE	VAL16	N-term	N-term	Yes
97.6	97.5	95.8	50.7	MET1	VAL3	N-term	N-term	Yes
95.5	87.9	86.6	53.5	MET1	PHE4	N-term	N-term	Yes
39.4	21.5	22.8	11.9	MET1	MET5	N-term	N-term	Yes
72.8	90.4	70.7	0.6	MET1	ALA11	N-term	N-term	Yes
31.8	58.4	45.9	0.4	MET1	VAL15	N-term	N-term	Yes
19.3	2.5	28.5	0.7	MET1	VAL16	N-term	N-term	Yes
18.7	0.0	34.5	0.0	MET1	ALA17	N-term	N-term	Yes
9.1	0.4	42.1	0.2	MET1	ALA18	N-term	N-term	Yes
5.7	0.0	3.1	0.8	MET1	ALA19	N-term	N-term	Yes
0.0	12.2	0.0	0.1	MET1	ALA53	N-term	N-term	Yes
99.8	99.8	99.8	96.2	VAL3	PHE4	N-term	N-term	Yes
0.3	0.2	0.3	11.2	VAL3	MET5	N-term	N-term	Yes
14.1	4.6	0.0	0.0	VAL3	VAL15	N-term	N-term	Yes
14.5	0.7	10.4	0.0	VAL3	VAL16	N-term	N-term	Yes
13.1	0.2	1.1	0.4	VAL3	ALA17	N-term	N-term	Yes
1.8	0.0	7.9	0.0	VAL3	ALA18	N-term	N-term	Yes
6.2	1.9	0.0	0.0	VAL3	VAL52	N-term	N-term	Yes
0.0	11.0	0.0	1.7	VAL3	ALA53	N-term	N-term	Yes
99.4	99.4	99.5	94.2	PHE4	MET5	N-term	N-term	Yes
0.0	0.0	1.5	18.1	PHE4	LEU8	N-term	N-term	Yes
8.8	0.3	2.4	4.2	PHE4	ALA11	N-term	N-term	Yes
6.9	9.0	0.0	0.0	PHE4	VAL15	N-term	N-term	Yes
17.2	0.0	3.5	7.6	PHE4	VAL16	N-term	N-term	Yes
10.6	0.2	0.5	0.8	PHE4	ALA17	N-term	N-term	Yes
6.7	0.5	10.1	1.6	PHE4	ALA18	N-term	N-term	Yes
11.8	0.0	0.1	0.5	PHE4	ALA19	N-term	N-term	Yes
0.7	0.7	0.0	9.6	PHE4	ALA53	N-term	N-term	Yes
16.2	10.5	6.6	30.1	MET5	LEU8	N-term	N-term	Yes
2.5	0.1	3.4	0.3	MET5	ALA11	N-term	N-term	Yes
0.5	0.0	0.8	3.9	MET5	VAL16	N-term	N-term	Yes
0.0	0.0	0.0	14.0	MET5	VAL48	N-term	N-term	Yes
0.0	0.2	0.0	5.4	MET5	ALA53	N-term	N-term	Yes
0.1	0.0	0.4	22.3	LEU8	VAL16	N-term	N-term	Yes
0.0	0.0	0.0	7.7	LEU8	ALA27	N-term	N-term	Yes
0.0	0.0	0.0	5.8	LEU8	VAL48	N-term	N-term	Yes
0.3	0.0	0.0	4.9	LEU8	VAL49	N-term	N-term	Yes
10.3	0.0	0.0	0.0	LEU8	ALA56	N-term	N-term	Yes
34.3	24.8	60.9	42.3	ALA11	VAL15	N-term	N-term	Yes
4.3	15.7	12.5	82.7	ALA11	VAL16	N-term	N-term	Yes
0.0	0.0	3.0	3.6	ALA11	ALA18	N-term	N-term	Yes
96.2	96.1	95.3	88.3	VAL15	VAL16	N-term	N-term	Yes
7.4	81.4	29.9	73.2	VAL15	ALA17	N-term	N-term	Yes
0.0	67.1	19.6	44.4	VAL15	ALA18	N-term	N-term	Yes

0.0	2.9	4.7	3.7	VAL15	ALA19	N-term	N-term	Yes
0.0	0.0	7.6	0.0	VAL15	ALA30	N-term	N-term	Yes
0.0	5.7	0.0	0.0	VAL15	VAL37	N-term	N-term	Yes
0.0	15.4	0.0	0.0	VAL15	ALA53	N-term	N-term	Yes
0.0	10.4	0.0	0.0	VAL15	ALA56	N-term	N-term	Yes
97.5	99.1	98.4	99.5	VAL16	ALA17	N-term	N-term	Yes
16.0	5.3	0.1	0.3	VAL16	ALA18	N-term	N-term	Yes
0.3	9.0	15.4	2.5	VAL16	ALA19	N-term	N-term	Yes
0.0	0.2	0.0	7.1	VAL16	VAL48	N-term	N-term	Yes
93.2	89.0	95.4	85.8	ALA17	ALA18	N-term	N-term	Yes
3.5	7.9	5.2	36.9	ALA17	ALA19	N-term	N-term	Yes
0.0	5.2	0.0	0.0	ALA17	VAL37	N-term	N-term	Yes
0.0	4.2	1.3	0.0	ALA17	ALA53	N-term	N-term	Yes
0.0	12.9	0.0	0.0	ALA17	ALA56	N-term	N-term	Yes
98.4	94.8	97.1	84.7	ALA18	ALA19	N-term	N-term	Yes
0.0	10.1	0.0	1.6	ALA18	VAL52	N-term	N-term	Yes
0.0	17.2	0.0	2.0	ALA18	ALA53	N-term	N-term	Yes
0.0	13.7	0.0	0.0	ALA18	ALA56	N-term	N-term	Yes
0.0	7.9	0.0	0.0	ALA19	VAL52	N-term	N-term	Yes
98.0	98.6	98.5	96.4	VAL26	ALA27	N-term	N-term	Yes
1.6	0.2	7.3	4.4	VAL26	ALA29	N-term	N-term	Yes
2.0	0.5	10.0	5.1	VAL26	ALA30	N-term	N-term	Yes
13.3	0.0	39.0	0.5	VAL26	VAL37	N-term	N-term	Yes
17.9	0.0	1.4	9.5	VAL26	LEU38	N-term	N-term	Yes
0.3	0.0	3.0	5.8	VAL26	VAL40	N-term	N-term	Yes
3.8	0.1	2.2	0.2	VAL26	VAL48	N-term	N-term	Yes
2.2	0.0	8.4	0.0	VAL26	VAL49	N-term	N-term	Yes
0.9	6.4	8.5	0.1	VAL26	VAL52	N-term	N-term	Yes
24.4	5.8	16.4	25.9	ALA27	ALA29	N-term	N-term	Yes
0.6	0.8	4.6	13.1	ALA27	ALA30	N-term	N-term	Yes
1.4	0.0	3.0	2.7	ALA27	VAL37	N-term	N-term	Yes
16.3	0.0	0.0	4.5	ALA27	LEU38	N-term	N-term	Yes
4.7	0.5	0.9	5.5	ALA27	VAL52	N-term	N-term	Yes
96.0	97.9	96.6	94.5	ALA29	ALA30	N-term	N-term	Yes
0.0	0.0	0.1	11.4	ALA29	VAL37	N-term	N-term	Yes
0.2	0.0	0.0	10.7	ALA29	LEU38	N-term	N-term	Yes
0.0	10.8	0.0	0.4	ALA29	VAL52	N-term	N-term	Yes
0.0	7.6	0.0	0.0	ALA29	ALA53	N-term	N-term	Yes
0.0	0.0	0.0	5.9	ALA30	VAL49	N-term	N-term	Yes
77.7	99.3	98.9	98.0	VAL37	LEU38	N-term	N-term	Yes
6.3	3.6	0.0	1.8	VAL37	POS39	N-term	N-term	Yes
73.2	52.7	57.2	9.7	VAL37	VAL40	N-term	N-term	Yes
27.5	53.5	42.4	0.0	VAL37	VAL48	N-term	N-term	Yes
35.7	56.9	42.4	0.0	VAL37	VAL49	N-term	N-term	Yes
2.3	6.8	9.1	0.0	VAL37	VAL52	N-term	N-term	Yes
98.4	99.8	99.7	95.3	LEU38	POS39	N-term	N-term	Yes
13.4	19.8	28.8	21.4	LEU38	VAL40	N-term	N-term	Yes
33.8	80.2	55.3	0.0	LEU38	VAL48	N-term	N-term	Yes
2.5	2.4	1.7	0.0	LEU38	VAL49	N-term	N-term	Yes
73.7	44.6	50.6	76.1	POS39	VAL40	N-term	N-term	Yes
4.8	0.8	2.5	0.1	POS39	VAL48	N-term	N-term	Yes
4.9	3.7	32.5	0.3	VAL40	VAL48	N-term	N-term	Yes

43.1	79.2	70.6	0.3	VAL40	VAL49	N-term	N-term	Yes
0.0	6.7	1.5	0.0	VAL40	VAL52	N-term	N-term	Yes
97.2	92.4	97.1	95.6	VAL48	VAL49	N-term	N-term	Yes
1.0	0.0	9.6	2.3	VAL48	VAL52	N-term	N-term	Yes
0.9	0.0	8.5	2.8	VAL48	ALA53	N-term	N-term	Yes
4.6	15.6	38.4	46.7	VAL49	VAL52	N-term	N-term	Yes
6.5	0.2	3.4	7.8	VAL49	ALA53	N-term	N-term	Yes
98.8	98.7	98.7	75.4	VAL52	ALA53	N-term	N-term	Yes
8.2	47.8	41.8	2.3	VAL52	VAL55	N-term	N-term	Yes
0.3	15.4	37.9	0.2	VAL52	ALA56	N-term	N-term	Yes
11.7	5.1	17.9	1.2	ALA53	VAL55	N-term	N-term	Yes
0.1	39.2	39.5	0.0	ALA53	ALA56	N-term	N-term	Yes
98.2	98.4	93.3	99.6	VAL55	ALA56	N-term	N-term	Yes
40.3	60.8	51.7	71.3	VAL63	VAL66	NAC	NAC	Yes
0.7	0.0	8.7	6.0	VAL63	ALA69	NAC	NAC	Yes
0.0	0.0	3.1	14.6	VAL63	VAL71	NAC	NAC	Yes
5.2	0.0	6.8	76.6	VAL63	VAL74	NAC	NAC	Yes
0.0	17.7	4.5	0.0	VAL63	ALA76	NAC	NAC	Yes
3.0	51.4	1.6	0.0	VAL63	VAL77	NAC	NAC	Yes
0.0	43.9	6.1	0.0	VAL63	ALA78	NAC	NAC	Yes
24.7	1.7	6.6	2.4	VAL66	ALA69	NAC	NAC	Yes
0.3	0.0	4.3	2.5	VAL66	VAL71	NAC	NAC	Yes
8.9	4.1	0.9	14.0	VAL66	VAL74	NAC	NAC	Yes
27.0	8.9	2.0	0.0	VAL66	ALA76	NAC	NAC	Yes
21.4	14.2	3.2	0.0	VAL66	VAL77	NAC	NAC	Yes
4.5	30.3	37.2	0.0	VAL66	ALA78	NAC	NAC	Yes
0.1	0.0	18.6	0.2	VAL66	VAL82	NAC	NAC	Yes
0.1	0.0	19.9	0.0	VAL66	ILE88	NAC	NAC	Yes
0.2	0.0	7.7	0.0	VAL66	ALA89	NAC	NAC	Yes
90.4	98.6	91.7	98.2	ALA69	VAL70	NAC	NAC	Yes
0.1	0.0	4.2	2.2	ALA69	VAL71	NAC	NAC	Yes
13.3	4.4	58.6	73.2	ALA69	VAL74	NAC	NAC	Yes
37.0	16.3	1.7	0.1	ALA69	ALA76	NAC	NAC	Yes
23.5	5.3	41.7	70.7	ALA69	VAL77	NAC	NAC	Yes
0.9	8.5	2.0	34.8	ALA69	ALA78	NAC	NAC	Yes
0.0	0.1	0.0	65.3	ALA69	VAL82	NAC	NAC	Yes
5.0	0.0	0.0	0.0	ALA69	PHE94	NAC	NAC	Yes
99.8	99.8	98.9	98.9	VAL70	VAL71	NAC	NAC	Yes
0.7	31.8	11.8	4.0	VAL70	VAL74	NAC	NAC	Yes
0.0	0.0	10.6	0.4	VAL70	ALA76	NAC	NAC	Yes
0.0	0.0	31.3	13.5	VAL70	VAL77	NAC	NAC	Yes
0.1	0.1	0.4	58.4	VAL70	VAL82	NAC	NAC	Yes
0.0	0.0	0.6	6.2	VAL70	ALA85	NAC	NAC	Yes
10.9	0.0	0.0	0.0	VAL70	PHE94	NAC	NAC	Yes
21.9	46.9	35.5	79.0	VAL71	VAL74	NAC	NAC	Yes
12.4	0.0	0.0	0.0	VAL71	PHE94	NAC	NAC	Yes
72.6	0.1	7.1	8.9	VAL74	VAL77	NAC	NAC	Yes
13.5	0.0	3.7	0.9	VAL74	ALA78	NAC	NAC	Yes
83.3	95.6	89.4	97.9	ALA76	VAL77	NAC	NAC	Yes
2.8	0.1	3.5	1.0	ALA76	ALA78	NAC	NAC	Yes
8.0	0.0	0.0	0.0	ALA76	ILE88	NAC	NAC	Yes
12.0	0.0	0.0	0.0	ALA76	ALA90	NAC	NAC	Yes

10.6	0.5	0.0	0.0	ALA76	ALA91	NAC	NAC	Yes
90.6	99.3	94.4	97.8	VAL77	ALA78	NAC	NAC	Yes
0.0	0.0	0.0	61.3	VAL77	VAL82	NAC	NAC	Yes
25.4	0.0	1.2	2.8	ALA78	VAL82	NAC	NAC	Yes
6.3	0.0	0.0	0.0	ALA78	ILE88	NAC	NAC	Yes
14.2	0.0	0.0	0.0	ALA78	ALA91	NAC	NAC	Yes
11.2	15.7	5.4	1.0	VAL82	ALA85	NAC	NAC	Yes
2.8	5.2	2.5	0.9	VAL82	ILE88	NAC	NAC	Yes
15.4	13.4	15.9	0.0	VAL82	ALA91	NAC	NAC	Yes
0.2	13.5	0.0	0.0	VAL82	PHE94	NAC	NAC	Yes
33.5	8.9	4.8	26.3	ALA85	ILE88	NAC	NAC	Yes
10.3	6.1	2.2	0.0	ALA85	ALA91	NAC	NAC	Yes
0.0	0.0	6.4	23.0	ALA85	VAL95	NAC	NAC	Yes
99.1	98.1	99.4	98.9	ILE88	ALA89	NAC	NAC	Yes
69.9	63.6	72.4	5.3	ILE88	ALA90	NAC	NAC	Yes
60.4	52.6	70.5	2.8	ILE88	ALA91	NAC	NAC	Yes
0.4	17.6	0.0	22.9	ILE88	PHE94	NAC	NAC	Yes
0.1	5.1	1.0	63.1	ILE88	VAL95	NAC	NAC	Yes
98.5	95.0	99.2	97.7	ALA89	ALA90	NAC	NAC	Yes
3.4	14.0	2.3	85.8	ALA89	ALA91	NAC	NAC	Yes
10.5	5.2	2.6	0.3	ALA89	PHE94	NAC	NAC	Yes
6.3	8.6	0.3	0.1	ALA89	VAL95	NAC	NAC	Yes
97.7	92.4	97.4	99.3	ALA90	ALA91	NAC	NAC	Yes
9.7	7.3	1.9	52.7	ALA90	PHE94	NAC	NAC	Yes
13.2	10.8	0.2	12.4	ALA90	VAL95	NAC	NAC	Yes
7.7	4.5	1.7	5.7	ALA91	PHE94	NAC	NAC	Yes
4.9	0.3	4.6	0.5	ALA91	VAL95	NAC	NAC	Yes
97.2	94.9	96.8	95.3	PHE94	VAL95	NAC	NAC	Yes
0.1	1.6	0.1	7.5	LEU100	ALA107	C-term	C-term	Yes
99.8	99.8	99.8	99.8	ALA107	PRO108	C-term	C-term	Yes
7.4	3.5	52.8	6.2	ALA107	ILE112	C-term	C-term	Yes
9.3	4.0	17.1	10.8	PRO108	ILE112	C-term	C-term	Yes
6.3	0.0	4.5	2.4	PRO108	LEU113	C-term	C-term	Yes
94.5	96.4	98.7	99.6	ILE112	LEU113	C-term	C-term	Yes
4.5	0.5	2.5	2.3	ILE112	MET116	C-term	C-term	Yes
1.4	7.5	22.6	4.8	LEU113	MET116	C-term	C-term	Yes
4.8	2.0	16.6	3.2	LEU113	PRO117	C-term	C-term	Yes
1.1	0.1	7.0	4.4	LEU113	VAL118	C-term	C-term	Yes
99.8	99.8	99.8	99.8	MET116	PRO117	C-term	C-term	Yes
28.5	8.9	0.8	12.7	MET116	VAL118	C-term	C-term	Yes
96.0	97.7	98.8	98.2	PRO117	VAL118	C-term	C-term	Yes
8.8	9.5	7.1	5.5	VAL118	PRO120	C-term	C-term	Yes
0.0	15.3	0.0	0.0	VAL118	TYR125	C-term	C-term	Yes
0.0	36.4	0.0	0.0	VAL118	TYR133	C-term	C-term	Yes
0.1	13.7	1.2	0.0	VAL118	TYR136	C-term	C-term	Yes
0.8	10.3	0.0	0.7	PRO120	TYR125	C-term	C-term	Yes
95.7	97.7	97.9	82.1	ALA124	TYR125	C-term	C-term	Yes
5.5	0.3	0.0	0.3	ALA124	MET127	C-term	C-term	Yes
0.1	9.8	0.0	0.0	ALA124	TYR133	C-term	C-term	Yes
0.0	98.2	27.1	10.6	ALA124	TYR136	C-term	C-term	Yes
51.8	46.1	6.6	13.7	TYR125	MET127	C-term	C-term	Yes
44.1	38.8	8.0	10.0	TYR125	PRO128	C-term	C-term	Yes

16.3	0.0	0.6	0.4	TYR125	TYR133	C-term	C-term	Yes
0.5	5.7	24.2	12.3	TYR125	TYR136	C-term	C-term	Yes
0.0	20.5	28.1	62.2	TYR125	PRO138	C-term	C-term	Yes
99.8	99.8	99.8	99.8	MET127	PRO128	C-term	C-term	Yes
63.0	14.2	99.0	95.5	MET127	TYR133	C-term	C-term	Yes
1.5	2.7	20.8	9.9	MET127	TYR136	C-term	C-term	Yes
0.0	7.0	1.7	0.1	MET127	PRO138	C-term	C-term	Yes
36.1	0.0	0.9	0.6	PRO128	TYR133	C-term	C-term	Yes
5.4	12.2	12.3	18.0	TYR133	TYR136	C-term	C-term	Yes
3.1	3.3	0.6	0.0	TYR133	PRO138	C-term	C-term	Yes
55.6	9.7	25.9	13.2	TYR136	PRO138	C-term	C-term	Yes
10.1	10.8	10.5	7.1	PRO138	ALA140	C-term	C-term	Yes
0.0	5.1	0.0	0.0	ALA19	ALA91	N-term	NAC	No
0.0	0.0	0.0	8.6	LEU38	VAL66	N-term	NAC	No
0.0	0.0	0.0	16.7	VAL40	VAL66	N-term	NAC	No
7.5	0.0	0.9	0.1	VAL52	VAL63	N-term	NAC	No
8.5	3.6	0.2	0.1	VAL52	VAL66	N-term	NAC	No
5.0	0.6	0.0	0.0	VAL52	VAL77	N-term	NAC	No
6.2	0.0	0.0	0.0	VAL52	ALA85	N-term	NAC	No
26.7	0.0	0.6	0.0	ALA53	VAL63	N-term	NAC	No
16.1	0.6	0.0	0.0	ALA53	VAL66	N-term	NAC	No
61.2	33.0	0.0	87.2	VAL55	VAL63	N-term	NAC	No
6.1	5.6	0.0	14.7	VAL55	VAL66	N-term	NAC	No
0.0	0.0	0.5	12.8	VAL55	VAL71	N-term	NAC	No
3.1	0.0	6.5	16.5	VAL55	VAL74	N-term	NAC	No
0.0	9.7	0.0	8.1	VAL55	ALA76	N-term	NAC	No
4.7	5.3	0.4	1.2	VAL55	VAL77	N-term	NAC	No
1.9	15.9	0.0	0.1	VAL55	ALA78	N-term	NAC	No
0.8	3.2	0.0	2.3	VAL55	VAL82	N-term	NAC	No
0.0	0.0	0.0	5.5	ALA56	VAL71	N-term	NAC	No
0.0	6.6	0.0	0.0	ALA56	VAL82	N-term	NAC	No
0.0	0.0	0.0	24.9	ALA85	LEU100	NAC	C-term	No
0.0	3.7	0.6	24.8	ILE88	LEU100	NAC	C-term	No
0.0	1.4	0.0	8.0	ALA89	LEU100	NAC	C-term	No
0.0	4.1	0.7	15.3	ALA90	LEU100	NAC	C-term	No
0.0	6.9	0.0	12.7	PHE94	LEU100	NAC	C-term	No
0.2	31.7	5.7	48.8	VAL95	LEU100	NAC	C-term	No
5.4	0.0	0.0	0.0	VAL95	ILE112	NAC	C-term	No
5.2	0.0	0.0	0.0	VAL95	LEU113	NAC	C-term	No
